# The cognitive side of communication in social insects

**DOI:** 10.1016/j.tics.2025.08.005

**Published:** 2025-11

**Authors:** Martin Giurfa

**Affiliations:** 1 Sorbonne Université, CNRS, Inserm, Institut de Biologie Paris-Seine, IBPS, F-75005 Paris, France; 2 Sorbonne Université, CNRS, Inserm, Centre de Neuroscience Neuro-SU, F-75005 Paris, France

**Keywords:** communication, cognition, pheromones, dance behavior, social insects

## Abstract

Social insects rely on multiple communication channels. These channels have traditionally been considered innate, eliciting stereotyped responses. However, recent research has shown that cognitive modulation occurs in communication contexts long assumed to be entirely genetically encoded, thus revealing a previously unrecognized cognitive plasticity in social insect communication.

## Challenging the innate perspective on insect communication

Animal communication is the process by which an individual (the sender) transmits a signal to another (the receiver), eliciting a change in the receiver’s behavior or internal state, typically in an adaptive manner for one or both. In social insects – including bees, wasps, ants, and termites – sophisticated, multi-channel communication systems underpin their complex social organization. Traditionally, these communication channels have been considered largely innate, eliciting stereotyped responses. However, research has begun to challenge this long-standing assumption. Communication channels in social insects are more flexible than previously thought and can be modulated by experience, revealing an unexpected role for cognitive processes within these systems. Here, I synthesize emerging evidence for this cognitive modulation, focusing on pheromones and the iconic waggle dance of the honey bee *Apis mellifera*.

## Pheromones as cognitive agents

Pheromones constitute a widespread communication channel among insects. They are chemical messengers, defined as substances secreted externally by an individual and received by another individual of the same species, in which they trigger specific behavioral or physiological responses [1]. Traditionally, pheromones have been considered stereotyped communication signals, having evolved to convey specific messages to conspecifics in a highly consistent and reliable manner.

Nevertheless, recent studies in bees and ants suggest that pheromones are not merely passive carriers of information, but can act as cognitive modulators, influencing responsiveness to reinforcers and the processes of learning and memory. These studies expose insects to pheromones of different valence (appetitive or aversive) and test whether such exposure, even after the pheromone is no longer present, alters responses to rewards or punishments, and affects learning and memory. Exposure to certain pheromones can indeed alter an individual's sensitivity to natural reinforcers such as reward or punishment. In the Argentine ant *(Linepithema humile)*, for instance, exposure to (Z)-9-hexadecenal – a trail pheromone component that induces trail-following behavior – increases acceptance of sucrose solutions of varying concentrations [2], indicating a change in the ants' subjective evaluation of food rewards induced by the pheromone. Similarly, in honey bees, exposure to geraniol – a major component of the Nasonov gland pheromone promoting attraction to food sources or nest entrances [3] – increases sucrose responsiveness [4]. This is measured using the proboscis extension response (PER) to a gradient of sucrose concentrations, demonstrating that geraniol enhances sensitivity to lower reward intensities. By contrast, exposure to 2-heptanone – a pheromone signaling aversive events [3] – decreases sucrose responsiveness [4]. A similar pattern emerges when responsiveness to noxious stimuli is assessed using the sting extension response (SER) to increasing voltages of electric shock. Isopentyl acetate, a major component of the sting alarm pheromone, enhances shock responsiveness, while geraniol decreases it [5]. Overall, these findings demonstrate that certain pheromones modulate the perceived salience of reinforcers according to their valence, promoting either appetitive or aversive behaviors. When the valence of the pheromone and the reinforcer are congruent, responsiveness is enhanced. Conversely, when they are incongruent, responsiveness is reduced, suggesting that the pheromonal signal shifts attention away from reinforcers that are irrelevant in the context defined by the pheromone.

**Figure 1:**
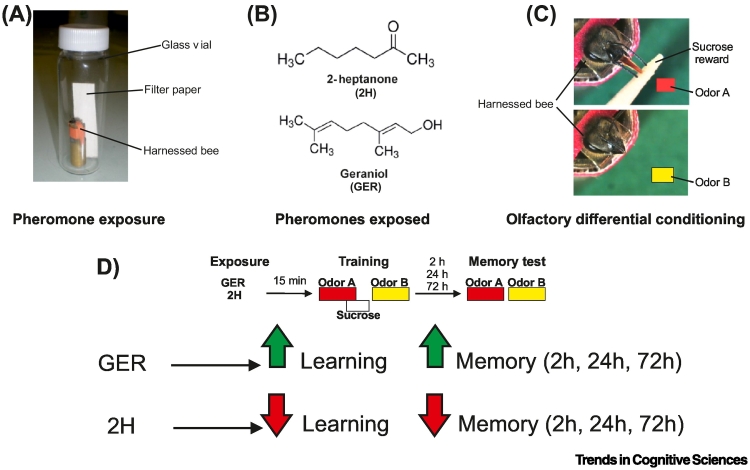
The cognitive effect of pheromones in bees. (A) A harnessed honey bee placed in a vertical tube with its head protruding is enclosed in a glass vial containing filter paper soaked with pheromone. (B) Two pheromones used for exposure: 2-heptanone (2H) – signaling aversive events, and geraniol (GER) – signaling appetitive events. (C) Olfactory conditioning of the proboscis extension response (PER) in honey bees. Harnessed bees learn to associate an odorant A with a sucrose solution, and a different odorant B with the absence of sucrose solution (differential olfactory conditioning). The conditioned response is the PER to the rewarded odorant. (D) Pheromones modulate olfactory learning and memory based on their valence. Upper part: experimental protocol. Lower part: results. Exposure to GER improved learning and memory retention with respect to control bees exposed to mineral oil, whereas 2H had a detrimental effect on both learning and memory retention compared with control bees [6].

Given their ability to modulate sensitivity to reinforcers, a natural next question is whether pheromones also influence associative learning and memory in a valence-dependent manner. This was studied in honey bees using a pheromone exposure combined with Pavlovian olfactory conditioning, where bees learned to associate one odor with a sucrose reward and another odor with its absence [6] (Figure 1). Results showed that geraniol and 2-heptanone respectively enhanced and impaired olfactory learning and memory. Crucially, these effects persisted over a long delay (e.g., 72 h post-conditioning), demonstrating that the cognitive modulation induced by pheromones has long-term consequences. When bees in an appetitive-searching state were exposed to geraniol, they learned and retained odor–sucrose associations more effectively. By contrast, exposure to 2-heptanone reduced bees’ propensity to learn about appetitive cues [6]. Importantly, this modulation occurred via aminergic circuits mediating motivational states, rather than primary odor processing pathways [6]. In ants, experiments on pheromone effects on associative learning have so far yielded negative results, unlike in honey bees. However, methodological factors – such as insufficient exposure time [7] or the use of operant contexts, where exploratory behavior may obscure learning-based decisions [2,7] – may explain the lack of observable effects.

These findings collectively indicate that, although pheromones evolved primarily as intraspecific chemical signals, they also exert a secondary, adaptive effect on the receiver’s cognitive state. This modulation of learning and memory, consistent with the pheromone’s valence, may have co-evolved with the primary signaling function to enhance behavioral flexibility and ecological relevance. Thus, pheromones in social insects are not merely chemical messengers but important cognitive agents modulating reinforcer responsiveness and associative learning.

## The waggle dance of honey bees: an innate behavior with room for cognition

Cognition also plays a role in communicative behaviors long considered purely innate. A unique example can be found in honey bees: the waggle dance [8], one of the most sophisticated systems for transferring information about appetitive rewards and the only known form of symbolic communication in an invertebrate. This dance is performed inside the hive by a forager returning from a profitable food source (>100 m). Distance is conveyed to followers via the dance tempo – either by the number of waggle runs per unit time or the duration of a single waggle run [8]. Direction is indicated by the orientation of the waggling straight portion between the two loops, relative to vertical, which serves as the azimuthal projection of the sun perceived en route to the goal [8]. This remarkable system, decoded by Nobel laureate Karl von Frisch, was long considered entirely innate. When introducing the dance, von Frisch famously stated: ‘The brain of a bee is the size of a grass seed and is not made for thinking. The actions of bees are mainly governed by instinct.’ [9]

However, recent research has demonstrated that cognitive modulation exists even within this highly structured behavior. While naive bees – isolated from the colony upon emergence – can perform the waggle dance and report food locations, their dances are significantly more disordered. They show greater angular divergence errors and incorrect distance encoding when lacking interaction with experienced foragers [10]. In normal colonies, workers transition to foraging with age, typically following waggle dancers around 8 days old and performing their first dances at approximately 12 days. Notably, exposure to experienced dancers improves the younger bees’ accuracy, particularly by reducing angular dispersion, though distance encoding remains distorted despite these interactions. These findings suggest that while the waggle dance is innately programmed, key features such as precision and efficiency can be modified through learning, revealing a cognitive component even in this emblematic instinctive communication [10].

The dance context also promotes olfactory learning in recruits following the dance inside the dark hive. Followers stay close to the dancer, making regular antennal contact to acquire spatial information. As the antennae are the main chemosensory organs of insects, this allows detection of odors on the dancer’s body, acquired from natural food sources. Dancers often pause to regurgitate nectar stored in their crop, offering it to followers via trophallaxis (mouth-to-mouth exchange). In this context, followers associate the body odor with the rewarding nectar, which is also scented. This associative learning has been demonstrated by capturing followers and testing their conditioned PER to the dancer’s odorants. These studies showed that, while following the dance, recruits form olfactory memories of nectar odors and later retrieve these memories when exposed to the same odors [11]. Thus, recruits leave the hive equipped with vectorial information about the food source location and with expectations of its olfactory signature. Flexibility is also visible in the decisions of dance followers: for instance, they may ignore dance vectors in favor of personal navigation memories [12] but rely more on dances when personal information becomes unreliable [13].

## Concluding remarks

These recent discoveries highlight that communication processes in social insects – long considered purely innate and characterized by rigid, stereotyped responses – are, in fact, subject to cognitive modulation. This may arise through specific agents (e.g., pheromones) or individual experience. In recent years, a notable shift has occurred in the study of social insect communication, with growing integration of cognitive perspectives. This shift reflects increasing recognition of the sophisticated cognitive abilities of bees and other social insects. These findings challenge long-standing assumptions about insect behavior and underscore the need to incorporate cognitive frameworks into studies of social communication. Recognizing the interplay between innate mechanisms and experience-based modulation opens new avenues for understanding the evolution and complexity of collective behavior in animal societies.
